# Bioelectrical Impedance among Rural Bangladeshi Women during Pregnancy and in the Postpartum Period

**DOI:** 10.3329/jhpn.v29i3.7871

**Published:** 2011-06

**Authors:** Saijuddin Shaikh, Kerry J. Schulze, Hasmot Ali, Alain B. Labrique, Abu Ahmed Shamim, Mahbubur Rashid, Sucheta Mehra, Parul Christian, Keith P. West

**Affiliations:** ^1^The JiVitA Project, Chalkmamrojpur, Shadullapur Road, Gaibandha, Bangladesh; ^2^Center for Human Nutrition, Department of International Health, Johns Hopkins Bloomberg School of Public Health, Baltimore, MD 21205, USA

**Keywords:** Bioelectrical impedance, Body composition, Phase angle, Pregnancy, Resistance, Bangladesh

## Abstract

Properties of bioelectrical impedance analysis (BIA) reflect body-composition and may serve as stand-alone indicators of maternal health. Despite these potential roles, BIA properties during pregnancy and lactation in rural South Asian women have not been described previously, although pregnancy and infant health outcomes are often compromised. This paper reports the BIA properties among a large sample of pregnant and postpartum women of rural Bangladesh, aged 12-46 years, participating in a substudy of a community-based, placebo-controlled trial of vitamin A or beta-carotene supplementation. Anthropometry and single frequency (50 kHz) BIA were assessed in 1,435 women during the first trimester (≤12 weeks gestation), in 1,237 women during the third trimester (32-36 weeks gestation), and in 1,141 women at 12-18 weeks postpartum. Resistance and reactance were recorded, and impedance and phase angle were calculated. Data were examined cross-sectionally to maximize sample-size at each timepoint, and the factors relating to BIA properties were explored. Women were typically young, primiparous and lacking formal education (22.2±6.3 years old, 42.2% primiparous, and 39.7% unschooled among the first trimester participants). Weight (kg), resistance (Ω), and reactance (Ω) were 42.1±5.7, 688±77, and 73±12 in the first trimester; 47.7±5.9, 646±77, and 64±12 in the third trimester; and 42.7±5.6, 699±79, and 72±12 postpartum respectively. Resistance declined with age and increased with body mass index. Resistance was higher than that observed in other, non-Asian pregnant populations, likely reflecting considerably smaller body-volume among Bangladeshi women. Resistance and reactance decreased in advanced stage of pregnancy as the rate of gain in weight increased, returning to the first trimester values by the three months postpartum. Normative distributions of BIA properties are presented for rural Bangladeshi women across a reproductive cycle that may be related to pregnancy outcomes and ultimately be used for assessing body-composition in this population.

## INTRODUCTION

Bioelectrical impedance analysis (BIA) is widely used in evaluating body-composition in epidemiological studies and clinical settings (1-4), usually by applying measures of resistance or impedance to population-specific, predictive equations for estimating total body-water (TBW), fat-free mass (FFM), and fat mass. Such equations have primarily been derived within well-nourished populations in developed-country settings (5-9). Application of BIA to determine body-composition in undernourished populations has been less common.

In South Asia, equations exist for predicting TBW in children aged 6-12 months (10) and school-age pre-adolescent Indian children (11,12) and fat mass in Indian men (13). The use of BIA for body-composition among South Asian women has been largely confined to groups of immigrants in other countries (5,14). Even fewer data are available des-cribing BIA properties during pregnancy or lactation in any South Asian populations, possibly due to uncertainty of the value of BIA in assessing the health or nutritional status in the absence of popu-lation-specific equations for body-composition (14). Additionally, complex relationships between intra and extra-cellular water and foetal and maternal nutrition and body mass compartments challenge the validity of BIA for estimating body-composition during pregnancy (15-17). Population studies of BIA, on the other hand, are emerging that describe distributions of resistance (R), reactance (Xc), and impedance (Z) in normal pregnancy in developed countries and reveal their potential to reflect risk of pregnancy-related complications. In Italian mothers, for example, Ghezzi *et al.* found that second trimester BIA indices were predictive of birthweight (17).

Maternal BIA could be particularly useful in South Asia where malnutrition and adverse pregnancy outcomes, including intrauterine growth retardation and preterm birth, are prevalent (18). Investigating relationships of pregnancy and health outcomes directly with BIA properties eliminates the need to derive estimates of body-composition. Further, BIA data obtained in large population studies could improve the understanding of the public-health use of BIA, especially where predictive equations based on sophisticated methods of body-composition measurement are unavailable.

The present study was designed to generate and compare normative cross-sectional distributions of bioelectrical impedance properties in early pregnancy, late pregnancy, and at three months postpartum in a cohort of women with viable pregnancies or live infants at the time of analysis in a typical rural setting in northern Bangladesh.

## MATERIALS AND METHODS

### Population and study design

This study was nested within a large randomized community-based trial evaluating vitamin A andbeta-carotene supplementation on all-cause, pregnancy-related maternal and infant mortality in northwestern Bangladesh during August 2001–February 2007 (19,20). Pregnancies were identified by registering married women of reproductive age and enrolling them into a five-weekly, home-based surveillance system, with a human chorionic gonadotrophin-based urine test confirming pregnancy among women reporting 30 consecutive amenstrual days. Data presented here were collected in a contiguous substudy area of 22 sq km with an estimated population of ~42,000, where pregnancies were enrolled and followed by the standard protocol but with additional clinical, anthropometric, biochemical and body-composition assessments done. Inclusion requirements for results reported here were provision of a valid BIA measurement obtained from women meeting the following criteria: (a) an early-pregnancy measurement taken within the first 12 weeks of gestation (first trimester); (b) a late-pregnancy measurement obtained between 32 and 36 weeks of gestation, inclusive (third trimester); or (c) a postpartum measurement obtained between 12 and 18 weeks postpartum, inclusive, among women with a living infant at the time of the visit. Women did not necessarily contribute data to all three time-points often due to their absence from home in the third trimester of pregnancy.

### Data-collection

Maternal weight and height were measured by trained and routinely-standardized anthropo-metrists. Weight with light clothing was measured at all visits on solar-powered SECA digital scales to the nearest 0.2 kg (SECA UNICEF Electronic Scale 890). Height was measured at the first trimester and three months postpartum visits to the nearest 0.1 cm using a portable Harpenden Pocket Stadio-meter (Cromwell, Crymch, UK), modified with a spirit-level affixed to the cross-bar to position subjects along the Frankfurt Plane. The mean of three readings was taken as the value for height. Intra and inter-worker technical standard deviations, expressed as percent coefficient of variation, were maintained at <1% of mean maternal height. Body mass index (BMI) was derived as weight/height^2^ (kg/m^2^). First-trimester height was used for calculating the BMI and BIA-related values at the third trimester, assuming negligible statural change during pregnancy.

Maternal age was recorded at the time of enrollment into the five-weekly pregnancy surveillance system, aided by a local and national events calendar, and updated for each subsequent visit by computer to account for time lapsed. Gestational ages at the time of the first and third trimester visits were calculated as the difference between the date of assessment and the recorded date of the last menstrual period (LMP), obtained at the enrollment visit based on the woman's recall, and checked for consistency against previous five-weekly surveillance-history data. The duration of time elapsed from birth to the time of the postpartum assessment was derived from the known date of birth.

Resistance (R) and reactance (Xc) were measured using single-frequency portable bioelectrical impedance analyzers (Quantum II, RJL system, MI, USA). Instruments were checked daily for accuracy, using a standard resistor. During the visit, women were seated at least 15 minutes while a health check-up was conducted, after which resistance and reactance were measured in women lying flat on a non-conductive surface following removal of shoes and jewelry (21). Displayed values were recorded when stabilized. 

Both resistance and reactance were expressed as denominators in relation to height-squared. These are conventional height-adjusted derivations of resistance and reactance used in prediction equations for body-composition. Thus, height^2^/R (cm^2^/Ω) and height^2^/Xc (cm^2^/Ω) were calculated for the first trimester and third trimester and for the three months postpartum time-point. Impedance (Z) and phase angle (PA) were calculated using the formulae: Z^2^=R^2^+Xc^2^ and PA=arctan (Xc/R) (22).  

### Statistical analysis

Statistical analysis was performed with the Stata software (version 10) (Stata Corporation, College Station, TX). Mean and standard deviations (SDs) were calculated to describe central tendencies and variability of data. Probability density plots were generated for resistance and reactance. Correlation analysis was used in evaluating associations of BMI with BIA measurements. The differences in cross-sectional BIA variable distributions were not tested across time-points due to lack of independence, although distributions of paired differences between the first and the third trimester, and the first or the third trimester and three months postpartum measures were tested against a null hypothesis of no change over time by analysis of variance (ANOVA) and paired *t*-test where longitudinal data were available. Within a cross-sectional visit, the differences in BIA variable distributions across maternal age-defined groups (<20, 20-29 and >30 years) were tested by ANOVA corrected by the Scheffe multiple comparison test (23). The statistical significance was set at p<0.05.

### Ethical approval

The Institutional Review Board of the Johns Hopkins Bloomberg School of Public Health in the USA and the Bangladesh Medical Research Council in Bangladesh approved the study. All women were consented by approved methods before participation.

## RESULTS

In total, 2,734 pregnancies were ascertained in the substudy area. Of these, 2,118 pregnant women consented and completed the first trimester visit, 1,338 completed the third trimester visit, and 1,602 completed the postpartum visit. After exclusions (e.g. BIA measurement not taken within timeframe for inclusion in the first or the third trimester or three months postpartum definitions) and losses to follow-up, 1,435, 1,237, and 1,141 women contributed data on BIA properties at the first trimester, third trimester, and three months postpartum respectively, for cross-sectional analyses at these times. Of those women, a large proportion contributed data at multiple time-points, such that 855 subjects contributed data both at the first and the third trimester, 757 at the first trimester and at three months postpartum, and 935 at the third trimester and three months postpartum.

Maternal supplementation with vitamin A, beta-carotene, or placebo had no effect on maternal measures of resistance (Ω) at the third trimester (placebo 647+80; vitamin A 641+73.3; beta-carotene 648+77; p=0.37) and three months postpartum (placebo 698+8; vitamin A 702+76; beta-caro-tene 695+82; p=0.40) visits. There were also no differences in reactance (Ω) in the third trimester (placebo 64.3+12.2; vitamin A 63.2+11.2; beta-caro-tene 63.6 +11.3; p=0.40) or three months postpartum (placebo 72.3+12.2; vitamin A 71.6+12.2; beta-carotene 71.4+12.7; p=0.60). The comparability of BIA distributions enabled the pooling of data across supplement groups for remaining analyses.

**Fig. 1.**
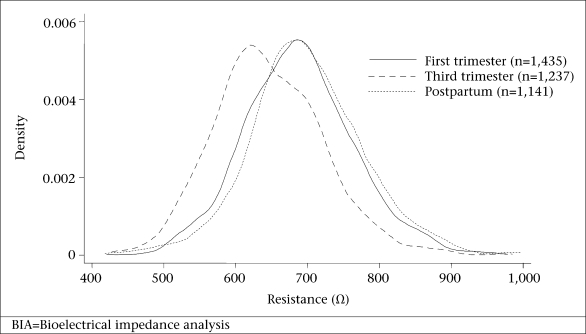
Probability density of resistance (Ω) measures (single frequency BIA with 50 kHz current) in first trimester, third trimester, and at three months postpartum among women of rural Bangladesh

**Fig. 2.**
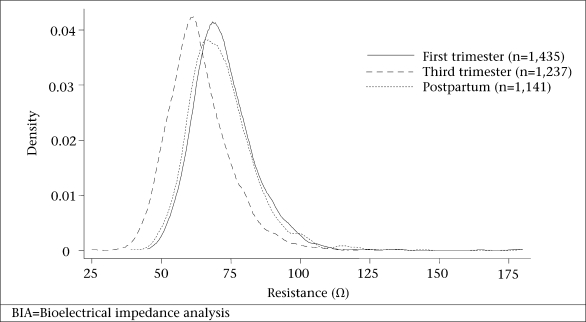
Probability density of reactance (Ω) measures (single frequency BIA with 50 kHz current) in the first trimester, third trimester, and at three months postpartum among women of rural Bangladesh

The demographic and socioeconomic characteristics are summarized in Table 1. The participants were mostly young adults aged 22.2+6.3 years at the time of their first trimester pregnancy ascertainment. At any given time point (because participants differed at first trimester, third trimester, and postpartum visits), 41-46% of the women were aged less than 20 years, 67-75% were in their first or second pregnancy, and 60-65% were not employed (Table 1). Anthropometric and BIA variables in Table 2 show that women were low in initial and postpartum weight (~42 kg), short in stature (~149 cm), and thin by BMI (~19 kg/m^2^). The average difference in weights between the first and the third trimester was 5.6 kg, comparable with an increment of 5.2+2.4 kg observed in the longitudinal cohort (p<0.001). Weight and BMI distributions by the third month postpartum were comparable with those in the first trimester. In the longitudinal cohort, the difference in weights from the first trimester to the third month postpartum was only 0.31+2.7 kg (p<0.01). The mean resistance and impedance were lower in the third trimester than the first trimester and only slightly higher at three months postpartum than during the first trimester. Reactance and phase angle were also lower in late pregnancy but were higher again at the postpartum period, similar to early pregnancy values (Table 2). Height^2^/R and height^2^/Xc increased from the first to the third trimester but also resembled early pregnancy by three months postpartum. The differences in the distributions of resistance and reactance by time of pregnancy visit are evident in Figure 1 and 2.

**Table 1.** Sociodemographic characteristics of rural Bangladeshi women by timing of bioelectrical impedance analysis during pregnancy and at postpartum

Characteristics	First trimester† (n=1,435)		Third trimester‡ (n=1,237)		Postpartum¶ (n=1,141)	
	No.	%	No.	%	No.	%
Age (years)*						
<20	592	41.3	564	45.7	525	46.1
20-29	643	44.8	556	45.0	504	44.2
≥30	199	13.9	115	9.3	110	9.7
Parity						
0	604	42.2	606	49.0	540	47.4
1	358	25.0	326	26.4	301	26.5
≥2	471	32.8	304	24.6	297	26.1
Education						
None	568	39.7	426	34.5	415	36.5
Primary	320	22.4	283	22.9	258	22.7
≥Secondary	544	38.0	527	42.6	465	40.8
Employed**						
Yes	577	40.3	451	36.5	402	35.3
No	856	59.7	785	63.5	737	64.7

*Age at pregnancy confirmation, first trimester;

†Missing values: age (n=1), parity (n=2), education (n=3), and occupation (n=2);

‡Missing values: age (n=2), parity (n=1), education (n=1), and occupation (n=1);

¶Missing values: age (n=2), parity (n=3), education (n=3), and occupation (n=2);

**Employment includes any work for which women were paid in cash or in kind, including self-employment and household-based economic activities

**Table 2.** Anthropometry and bioelectrical impedance values at first and third trimesters of pregnancy and postpartum*

Variable	First trimester† (n=1,435)	Third trimester‡ (n=1,237)	Postpartum¶ (n=1,141)
Weight (kg)	42.1±5.7	47.7±5.9	42.7±5.6
Height (cm)	149.4±5.2	NA	149.1±5.2
BMI (kg/m^2^)§	18.8±2.1	21.4±2.1	19.2±2.0
Resistance (Ω)	688±76.6	646±76.6	699±79.4
Reactance (Ω)	73±12.0	64±11.5	72±12.4
Impedance (Ω)	692±76.5	649±76.7	702±79.4
Phase angle (°)	6.1±1.1	5.7±0.9	5.9±1.1
Height^2^/R (cm^2^/Ω)§	32.9±4.5	35.1±4.9	32.3±4.6
Height^2^/Xc (cm^2^/Ω)§	315±54.2	362±69.6	319±57.1

*Values are mean±SD;

†Missing values: weight (n=6) and BMI (n=6);

‡Missing values: weight (n=16), BMI (n=22), height^2^/R (n=6), and height^2^/Xc (n=6);

¶Missing values: weight (n=13), BMI (n=13), reactance (n=1), impedance (n=1), phase angle (n=1), and height^2^/Xc (n=1);

§First trimester height used for calculating third trimester BMI, height^2^/R, and height^2^/Xc;

BMI=Body mass index;

NA=Not applicable;

R=Resistance;

SD=Standard deviation;

Xc=Reactance

BMI and resistance negatively covaried in the first trimester (r=-0.49, p<0.001). The relationship between BMI and reactance was also negative, although weaker (r=-0.22, p<0.001). Conversely, the correlation between BMI and height^2^/R was positive (r=0.43, p<0.001). Similar cross-sectional associations were present at the third trimester and three months postpartum.

Age was a determinant of bioelectrical impedance at each visit, with women aged less than 20 years (i.e. adolescents), having the most distinct characteristics (Table 3). Resistance and impedance werehigher in women aged less than 20 years than in older age-groups at each visit. Reactance was not associated with age but phase angle was consistently lower in adolescents compared to women aged 20-29 years. Height^2^/R was consistently lower in adolescents than in age-groups of 20-29 years and ≥30 years.

**Table 3.** Anthropometry and bioelectrical impedance values at first and third trimesters and postpartum by maternal age*

Variable	First trimester†			Third trimester‡			Postpartum¶		
	<20 years	20-29 years	>30 years	<20 years	20-29 years	>30 years	<20 years	20-29 years	>30 years
Weight (kg)	41.4±5.1**	42.8±6.0	42.2±6.3	46.8±5.4**	48.6±6.1	47.9±6.8	41.5±4.8††	43.9±6.1	43.1±5.8
Height (cm)	148.7±5.3**	149.9±5.2	149.7±4.8	NA	NA	NA	148.5±5.3**	149.7±5.2	149.4±4.4
BMI (kg/m^2^)^§^	18.7±1.9**	19.0±2.3	18.8±2.4	21.1±1.9**	21.6±2.2	21.4±2.6	18.8±1.7††	19.6±2.2	19.3±2.3
Resistance (Ω)	705±75.9^g^††	678±75.0	674±76.1	661±78.7††	635±70.8	618±75.4	713±80.8††	690±75.1	673±79.7
Reactance (Ω)	73±11.5	73±11.8	72±14.0	64±10.6	64.2±12.4	61±9.8	72±12.0	72±12.9	70±11.6
Impedance (Ω)	708±75.8††	682±74.9	678±76.0	664±78.8††	639±71.0	621±75.5	716±80.7††	693±75.1	677±79.4
Phase angle (°)	5.9±1.0**	6.2±1.1	6.1±1.3	5.5±0.9**	5.8±1.0	5.7±0.8	5.8±1.0**	6.0±1.1	6.0±1.2
Height^2^/R (cm^2^/Ω)§	31.8±4.2††	33.6±4.6	33.7±4.5	34.0±4.8††	35.9±4.6	36.8±5.1	31.4±4.5††	33±4.6	34±4.8
Height^2^/Xc (cm^2^/Ω)§	312±51.1	316±55.1	322±59.5	357±66.2^‡‡^	364±72.5	377±67.0	316±56.0	320±57.2	330±61.9

*Values are mean±SD; ANOVA with Scheffe multiple comparison test conducted within timepoints among age-groups;

†Missing values: weight (n=3) and BMI (n=3) for age <20 years; weight (n=2) and BMI (n=2) for age 20-29 years; weight (n=1) and BMI (n=1) for age ≥30 years;

‡Missing values: weight (n=6), BMI (n=8), height^2^/R (n=2), and height^2^/Xc (n=2) for age <20 years; weight (n=9), BMI (n=13), height^2^/R (n=4), and height^2^/Xc (n=4) for age 20-29 years; weight (n=1) and BMI (n=1) for age ≥30 years;

¶Missing values: weight (n=3), BMI (n=3), reactance (n=1), impedance (n=1), phase angle (n=1), and height^2^/Xc (n=1) for age <20 years; weight (n=8) and BMI (n=8) for age 20-29 years; weight (n=2) and BMI (n=2) for age ≥30 years;

§First-trimester height was used in calculating BMI, height^2^/R, and height^2^/Xc at third trimester;

**Women aged <20 years were significantly (p<0.05) different than women aged 20-29 years;

††Women aged <20 years were significantly (p<0.05) different than women aged 20-29 years and >30 years aged;

‡‡Women aged <20 years were significantly (p<0.05) different than women aged >30 years;

ANOVA=Analysis of variance;

BMI=Body mass index;

NA=Not available;

R=Resistance;

SD=Standard deviation;

Xc=Reactance

## DISCUSSION

This study is the first to report distributions of bioelectrical impedance properties from a rural population of pregnant and postpartum women in South Asia, typical for the region in being stunted and thin, poorly educated, and in experiencing first pregnancy at a young age. The distributions may be considered normative in that the measurements were from a population-based sample of women with a viable pregnancy or a live, breastfed infant at the time of respective measurements. BIA may be a particularly useful tool during pregnancy as it has safely been used for exploring distributions of bioelectrical properties, hydration status, and body-composition components in various studies during pregnancy (3,13-17). However, characterizing population-specific distributions of these properties is required to realize their potential for describing health implications for mothers and offspring.

Bioelectrical impedance is the degree to which a standard current, typically 50 kHz, is slowed (resistance) or stopped (reactance) as it passes through the body. Resistance is typically reduced by body-fluids, which are proportional to lean body-mass and is increased by body-fat, through which a current is not readily conducted. Reactance reflects cell-membrane capacitance. Resistance and reactance are vectors that are related by phase angle, for which a larger value reflects increased body-cell mass (24,25). However, these generalizations must be interpreted carefully within the population of interest as bioelectrical properties and their relationship to body-composition are affected by body-size, factors relating to health, nutrition, hydration, and stage of life.

As such, the magnitude of the values reported here for resistance and reactance among rural Bangladeshi women are higher than those observed in other non-Asian populations. The mean values of both the variables in the first and the third trimester were 688 and 73 Ω and of 646 and 64 Ω respectively, which are higher than those reported from two small studies in the United States (15,16) and a larger one in Italy (17) using similar single-frequency BIA techniques. In those investigations, the mean resistance and reactance values were 560-589 Ω and 49-69 Ω in the first trimester and 506-521 Ω and ~60 Ω in the third trimester respectively. During the postpartum period, the mean values in our study were nearly identical to the first trimester values (699 Ω and 72 Ω respectively), similar to the pattern observed in the American and Italian studies, where the mean postpartum resistance and reactance were 537-590 Ω and 67-69 Ω respectively (15-17).

A difference in body-volume, which is markedly smaller in South Asian than Western women, may be one explanation for inter-population differences in resistance (24,26). Estimated total body-volume of the American and Italian first-trimester gravida ~160 cm in height and ~60 kg in weight is ~56.4 L whereas the body-volume of the first-trimester Bangladeshi mothers is ~38.9 L, or approximately 30% lower (27). Higher resistance measures among the smaller Bangladeshi women are consistent with the fact that resistance is inversely proportional to the volume of the mass, or body, through which it is conducted (24).

The pattern of decline in resistance and reactance in late pregnancy and the upward shift at postpartum, in association with gain and loss in weight, have been observed elsewhere during pregnancy (15-17,28). Gains in body-weight during pregnancy distribute across the conceptus (foetus, placenta, and amniotic fluid), uterine and breast-tissue, body-fluid compartments, and maternal fat-tissues. In American and Swedish women, declines in pregnancy-associated resistance and reactance predicted gains in total body-water, which represented ~50% of total pregnancy-associated weight gain (15,16,29). It is likely that changes in resistance and reactance in Bangladeshi women also reflect pregnancy-associated alterations in total body-water.

The measured phase angle among Bangladeshi women during pregnancy also differed from values reported in non-Asian populations. Lukaski *et al*. reported a mean value of 6.6 degrees for phase angle in early pregnancy (12-14 weeks gestation) that remained unchanged throughout pregnancy into the postpartum months (16). In contrast, phase angle in the present study was 6.1 degrees in early pregnancy and declined to 5.7 degrees late in pregnancy, possibly indicating a more compromised body-cell mass profile at the outset of pregnancy and with advancing gestation among Bangladeshi women.

Among the participating women, resistance and reactance were lower, and height^2^/R was higher with increasing BMI. The direction of these cross-sectional associations is similar to those described in German adults with Western distributions of BMI (30,31). Future work will reveal whether this association is explained by increases in lean or fat-mass, both of which contribute to BMI. We also detected age-related differences in bioelectrical impedance variables that likely reflect maturational differences in body-composition between younger and older women. For example, resistance and impedance were higher and phase angle was generally lower among adolescent gravid compared to older pregnant women. These differences may reflect both smaller body-size and lower lean mass of younger women as they continue to grow towards peak stature and fat-free mass. Such maturational differences in resistance and reactance have been observed elsewhere (30-33). Other studies of the impact ofpregnancy and lactation on adolescent statural and lean body-growth in this population have shown that it is attenuated compared to that observed in non-pregnant young women of the same age (34). Continued assessment of our Bangladeshi mothers might reveal the full cost of early pregnancy and lactation on attained adult size and body-composition. Finally, declines in phase angle with advancing age have been observed here and elsewhere (30,35,36), and determining whether the extent of such declines is related to early or frequent childbearing in this nutritionally-challenging environment is of interest.

We observed distributions of resistance and reactance that are displaced upward compared to European and American populations but that exhibit qualitatively similar changes during pregnancy and at the postpartum period. Bioelectrical impedance distributions at three months postpartum suggest a return to an early-pregnancy body-composition by that time across a single reproductive cycle. Given the unique anthropometric characteristics of this population, population-specific prediction equations will be required to derive body-composition estimates in these women. We intend to further explore these BIA properties and subsequent body-composition measures in relation to maternal health, pregnancy outcomes, and infant health and survival.

## ACKNOWLEDGEMENTS

The study was funded by the Bill & Melinda Gates Foundation, Seattle, WA, USA (Grant No. 614 Global Control of Micronutrient Deficiency), US Agency for International Development, Washington, DC, USA (Global Research Activity GHS-A-00-03-00019-00), and Sight and Life Research Institute, Baltimore, MD, USA. The study was conducted under an agreement with the National Integrated Population and Health Programme of the Ministry of Health and Family Welfare of the Government of the People's Republic of Bangladesh. The contributions of the JiVitA field and data management teams and Johns Hopkins collaborators (Allan Massie, Maithilee Mitra, and Lee Wu) are gratefully acknowledged.
